# Supplementary material for: Switching of chiral magnetic skyrmions by picosecond magnetic field pulses via transient topological states
Source: Sci Rep. 2016 Jun 8;6:27146. doi: 10.1038/srep27146 (PMC4897691; doi:10.1038/srep27146)
Supplement: Supplementary Information [file srep27146-s1.pdf]

Supplemental Material for ”Switching of chiral magnetic skyrmions by picosecond magnetic field pulses via transient topological states”

Changhoon Heo[1,+], Nikolai S. Kiselev[2,+,*], Ashis Kumar Nandy[2,+], Stefan Blügel[2], and Theo Rasing[1]

[1]Radboud University, Institute for Molecules and Materials, Heyendaalseweg 135, 6525 AJ, Nijmegen, The Netherlands
[2]Peter Grünberg Institut and Institute for Advanced Simulation, Forschungszentrum Jülich and JARA, D-52425 Jülich, Germany
[+]these authors contributed equally to this work
[*]n.kiselev@fz-juelich.de

ABSTRACT

Details of the phase diagram calculation

In the main text, we presented the phase diagram of the ground state of an extended three monolayer thick film, see Fig. 1a. Here, we present the details of the calculations leading to the phase diagram as well as the dependence of the phase transition lines on the film thickness. To find the phase transition line between spin spiral (SS) and saturated ferromagnetic (FM) states we used the following scheme. We numerically calculated an equilibrium period and a corresponding energy for varying values of D/J at fixed K/J. The value at which the period of the SS tends to infinity and the energy difference between SS and FM states tends to zero, we identify as second order phase transition line. In Fig. 1 we show an example of such a calculation for $K/J = 0.4$. In particular, Fig. 1a shows the total energy density as a function of the SS period P_{SS} for different values of D/J. It is easy to show that in case of a thin film of a chiral magnet with a simple cubic structure and nearest-neighbor exchange and Dzyaloshinskii-Moriya interactions (DMI) the preferable direction for the SS propagation is $\langle 110 \rangle$, along the diagonal of a plane of the elementary cubic cell. The total energy density is calculated in unit of exchange coupling constant J. The position of the energy minimum for varying D/J denoted by the doted line in Fig. 1a corresponds to the equilibrium period of the SS state. As follows from Fig. 1a and b the equilibrium period runs to infinity between D/J=0.369 and 0.370 while the energy of SS tends to the energy of the FM state, which can be calculated precisely and for this case equals $-5.73(3)\ J$. In Fig. 1c we show the energy density difference between SS and FM states. The ΔE_{SS} tends to zero when one approaches the phase transition point at about $D/J = 0.3695 \pm 0.0005$. Such an approach allows to identify the phase transition point for D/J with a precision, which depends on the step size in D/J.

On the left side of the transition line in Fig. 1b, we also show the size dependence for the metastable isolated skyrmion (iSk). In Fig. 1c, the calculated energy of an iSk with respect to the FM state illustrates the metastability of iSk, since the energy of the iSk is always higher than the energy of FM state.

To find the dependence of the energy density on the period of the SS presented in Fig. 1a, we performed the following calculations. We calculated the energy of the SS state after a full relaxation on a domain with periodic boundary conditions (PBC). As an initial configuration we used a homogeneous SS with the period defined as $P_{\mathrm{SS}} = N/i$, where N is the size of the domain along $\langle 110 \rangle$ and i is a fixed integer number. Due to PBC, the period of the SS during the relaxation remains conserved. By varying the size of the simulated domain, one can find the energy dependence as a function of P_{SS}. Fig. 2 illustrates our approach for the case $K/J = 0.4$ and $D/J = 0.375$. Here we assume $i = 1$, the size of the domain corresponds to one SS period. Fig. 2a and b show the initial homogeneous and relaxed inhomogeneous SS states, respectively. Figs. 2c and d show relaxed SS for larger domains corresponding to larger SS periods. Compare the relaxed energies corresponding to Figs. 1b, c and d in this particular case, the lowest energy state is the SS state with period $P_{\mathrm{SS}} = \sqrt{2}a \cdot 19 = 26.87a$. The step in energy dependence on the period in such an approach is inversely proportional to the integer number of periods i in the domain, $\Delta P_{\mathrm{SS}} = \sqrt{2}a/i$. However, for higher precision it is important to use large $i \gtrsim 10$, when the equilibrium period of the spin spiral is incommensurate with the lattice constant a.

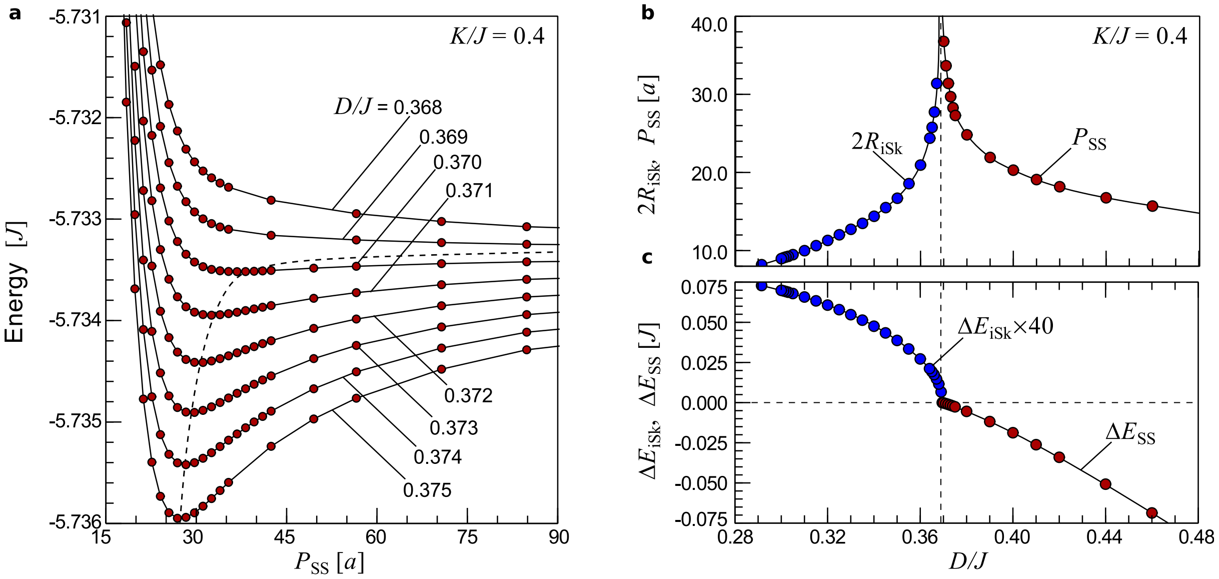

Figure 1. The scheme to determine the phase transition point for varying D/J at fixed K/J: (a) Energy density as a function of spin spiral (SS) period, P_{SS}, numerically calculated at zero applied field for model Hamiltonian Eq. 1 in the main text. Calculations are done for a fixed ratio $K/J = 0.4$, and varying values of D/J close to the phase transition into the saturated ferromagnetic state. Doted line follows the position of the energy minimum for each D/J. It approaches (converges to) the energy of the ferromagnetic state when D/J approaches the critical value. The period of SS is presented in the unit of crystal lattice parameter a. (b) The dependence of the SS period and the size of an isolated skyrmion on D/J for a fixed $K/J = 0.4$. Near the phase transition point, $P_{\mathrm{SS}} \to \infty$ and $D_{\mathrm{iSk}} \to \infty$. (c) The energy density for the SS state, ΔE_{SS}, and for the isolated skyrmion state, ΔE_{iSk}, relative to the saturated ferromagnetic state as function of D/J. The equilibrium skyrmion size calculated in square domain of $100 \times 100 \times 3$ spins with periodical boundary conditions in the xy-plane.

Note, there is an analytical solution for the phase transition line between ferromagnetic and spin spiral states derived by Dzyaloshinskii in the frame of micromagnetic continuum approximation.[1,2] However the approach of Dzyaloshinskii ignores the effect of magnetization modulation along the thickness. Thus, it is valid only for two limiting cases: pure two-dimensional case (single monolayer) and the bulk system where the effects of the chiral surface twist can be neglected. Recently, this effect and the role of magnetic modulation along the thickness of the magnetic layer has been studied in the context of skyrmion stability in cubic helimagnets.[3] It has been shown that such modulations are localized close to the free surfaces and sufficiently reduce the energy of the skyrmion state as well as lead to the stabilization of other earlier unknown localized magnetic states – *chiral bobbers*.

Iin both cases, the iSk and the spin spiral, we observe pronounced effects of modulations along the thickness in both cases of iSk and spin spiral. Fig. 2e shows the zoomed in area of the relaxed spin spiral state and illustrates the effect of chiral twist along the thickness of the layer, see small angle δ between spins in the middle layer and spins in top and bottom layers. A similar modulation is apparent in the spin structure of iSk.

Such modulations significantly reduce the energy of inhomogeneous states. In order to illustrate the pronounced effects of modulations on the stability of a spin spiral, we adopt an analytical solution derived by Dzyaloshinskii and compare the results with our numerical calculations. In particular, in the micromagnetic approach assuming small angles of rotation between nearest neighbor spins, the energy density of inhomogeneity for the spin spiral with period λ is

$$\Delta E = \frac{1}{\lambda}\int_0^\lambda \left[\mathscr{K}\cdot\sin^2\phi + \mathscr{J}\frac{\mathrm{d}\phi}{\mathrm{d}r}^2 - \mathscr{D}\frac{\mathrm{d}\phi}{\mathrm{d}r}\right]\mathrm{d}r, \tag{1}$$

where $\phi \equiv \phi(r)$ describes the polar angle of magnetization. For a simple cubic lattice, the micromagnetic constants in Eq. 1 are related to the constants of a discrete model via $\mathscr{J} = J/a$, and $\mathscr{D} = 2D/a^2$, $\mathscr{K} = K/a^3$. Minimization of Eq. 1 gives an expression for energy minimum $\Delta E_{\min}$ and an equilibrium period of the spin spiral $\lambda_{\min}$. Ref.[4] gives a simple and elegant proof for the following inequality

$$\Delta E_{\min} \geq 8\sqrt{\mathscr{K}\mathscr{J}} \pm 2\pi\mathscr{D}, \tag{2}$$

where the first term represents twice the energy of an ordinary Bloch wall, while the second one reflects the contribution of

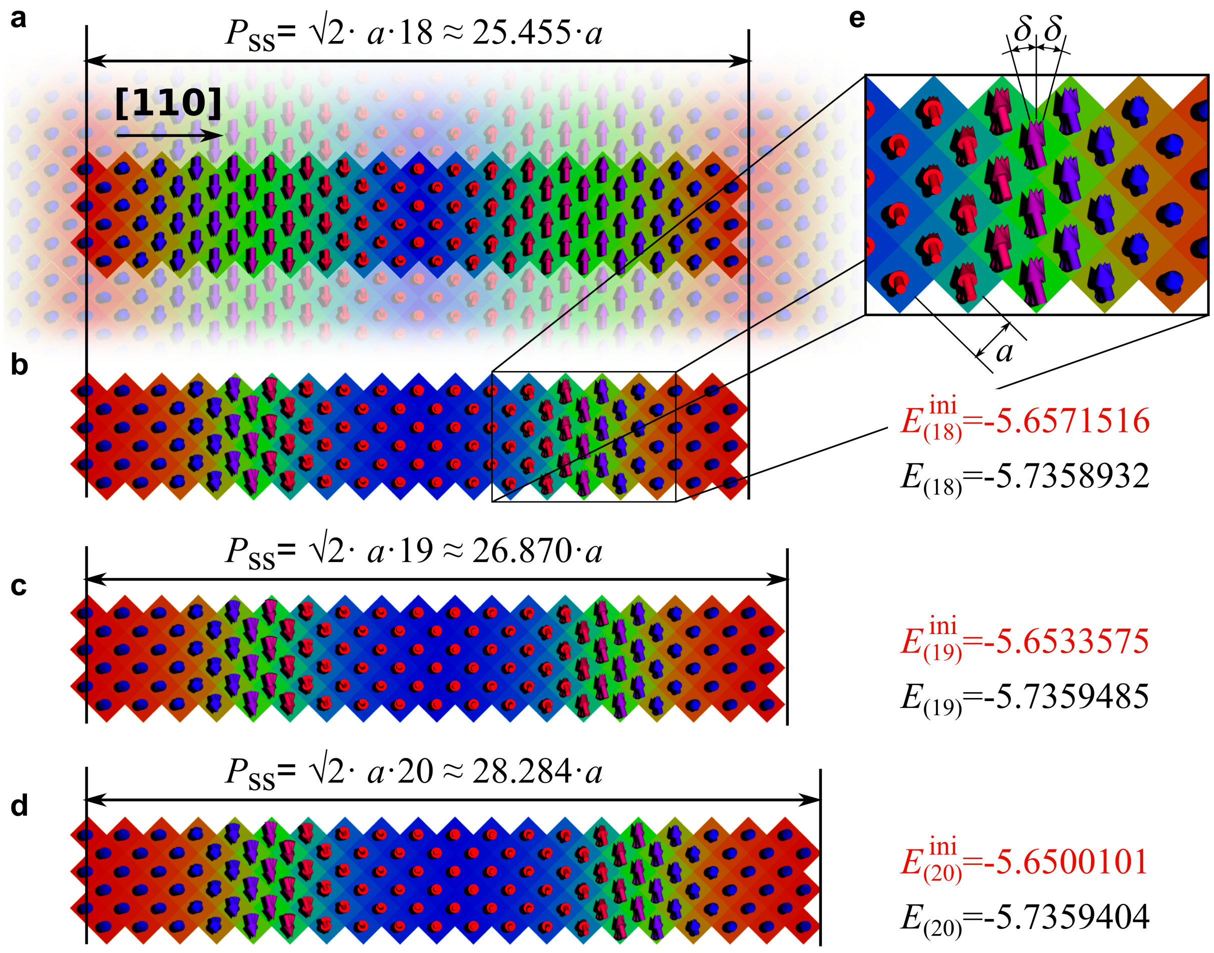

Figure 2. Snapshots of SS propagating along $\langle 110\rangle$ of a simple cubic lattice for different characteristic domain size defined as $\sqrt{2}\cdot a\cdot N$ along $\langle 110\rangle$: (a) Homogeneous SS used as the initial state for the following relaxation. Transparent spins indicate periodical boundary conditions used in the relaxation process. (b) Inhomogeneous SS after the relaxation of the initial state shown in a. (c)-(d) Inhomogeneous SS states after the relaxation for the characteristic domain size with $N = 19$ and 20, respectively. (e) Zoomed view of the inhomogeneous SS in b. The angles δ indicate a modulation of the spins along the thickness. $E^{\mathrm{ini}}_{(N)}$ (in red) indicates the energy density in the unit of J for the homogeneous initial SS state and $E_{(N)}$ (in black) indicates the energy density of the inhomogeneous fully relaxed SS state.

DMI. The sign of the last term in Eq. 2 depends on the sense of rotation of the spins in the spiral. If the value of $\Delta E_{\mathrm{min}} > 0$, the ground state corresponds to a collinear FM state, if $\Delta E_{\mathrm{min}} < 0$ one may conclude that there exists an inhomogeneous state, which has an energy lower than the FM state. Therefore, a criterion for appearance of an inhomogeneous spin spiral reads

$$\frac{\mathscr{D}}{\mathscr{J}} = \frac{4}{\pi}\sqrt{\frac{\mathscr{K}}{\mathscr{J}}}, \tag{3}$$

or in units of the discrete model for the case of a simple cubic lattice

$$\frac{D}{J} = \frac{2}{\pi}\sqrt{\frac{K}{J}}. \tag{4}$$

In Fig. 3a we present the analytical solution for the phase transition line (Eq. 4), see the dashed red line. The symbols in the figure represent our numerical calculations for different thicknesses of the film. As has been mentioned before, the micromagnetic approach is a good approximation for transitions in the case of one monolayer, see $L_n = 1$, open circles. The discrepancy appears only for the high anisotropy case, $K/J > 0.3$, see inset in Fig. 3a. This is because in the high anisotropy case, the approximation of small angle rotation fails. For any finite thickness $L_n > 1$, the transition always occurs below the

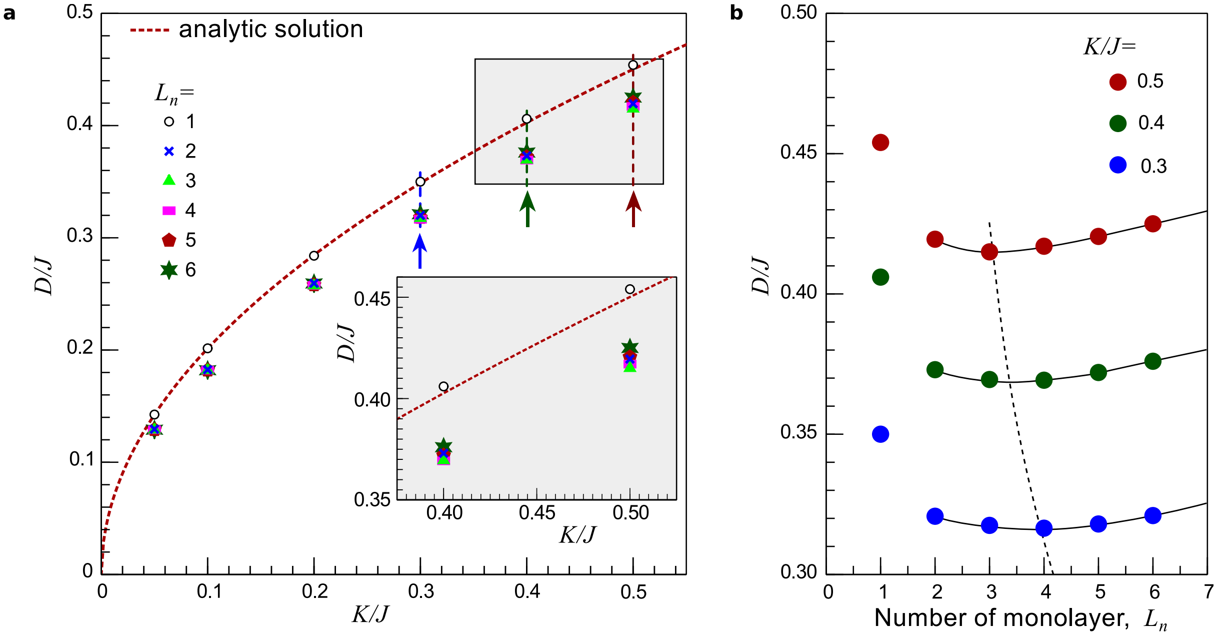

Figure 3. Phase transition of spin spiral state for different thicknesses: (a) Phase transition between spin spiral and ferromagnetic states calculated for different thicknesses of the (001) film of a simple cubic lattice. Dashed line is the analytical solution (Eq. 4) employing the micromagnetic approach. Here L_n denotes the thickness of the film in number of monolayers. Inset shows zoomed area marked as (gray) rectangle in the main figure. (b) Dependence of the critical values of D/J for the phase transition as a function of the magnetic film thickness L_n for fixed values of K/J marked by arrows in figure a.

transition corresponding to the single monolayer ($L_n = 1$). Indeed, Fig. 3b shows an abrupt change in the transition values of D/J between pure two-dimensional case, $L_n = 1$ and the one of finite thicknesses, $L_n \geq 2$. The transition value of D/J passes through a minima and then gradually increases. The critical thickness which corresponds to the minima strongly depends on anisotropy value, see the dashed line in Fig. 3b. For the bulk limit, the critical value of D/J gradually converges to the pure two-dimensional case of single monolayer. Such a dependence for the transition line on the thickness has to be taken into account for the calculation of the stability range for iSks.

Supplementary movie

The movie shows the back and forth skyrmion switching by the sequence of magnetic field pulses of amplitude B_0 = 3 T and width t_w = 15 ps with alternating polar angles $\theta = 45°$ and $\theta' = 135°$. For the simulations in a square shape domain of $100 \times 100 \times 3$ spins, we have used the material parameters $J = 5$ meV, $D/J = 0.16$, $K/J = 0.1$, $\alpha = 0.1$ and OBC. An interval of about 200 ps between two consecutive switching events suggests a skyrmion switching rate in the GHz range.

References

1. Dzyaloshinskii, I. E. Theory of helicoidal structures in antiferromagnets. III, *Sov. Phys. JETP* **20**, 665-668 (1965).
2. Izyumov, Y. A. Modulated, or long-periodic, magnetic structures of crystals. *Sov. Phys Usp.* **27**, 845 (1984).
3. Rybakov, F. N., Borisov, A. B., Blügel, S. & Kiselev, N. S. New type of stable particlelike states in chiral magnets. *Phys. Rev. Lett.* **115** 117201 (2015).
4. Bogdanov, A. N. & Yablonskii, D. A. Thermodynamically stable ”vortices” in magnetically ordered crystals. The mixed state of magnets. *Sov. Phys. JETP* **68**, 101-103 (1989).
